# Determinants of tobacco smoking among rural-to-urban migrant workers: a cross-sectional survey in Shanghai

**DOI:** 10.1186/s12889-015-1361-x

**Published:** 2015-02-12

**Authors:** Yao Liu, Huijiang Song, Tianying Wang, Tianhao Wang, Hua Yang, Jian Gong, Yao Shen, Wei Dai, Jing Zhou, Shanzhu Zhu, Zhigang Pan

**Affiliations:** Department of General Practice, Zhongshan Hospital Fudan University, 180 Fenglin Road, Shanghai, 20032 China; Department of General Practice, Sanlin Community Health Service Center, 375 Sanlin Road, Shanghai, 200124 China

**Keywords:** Rural-to-urban migrants, Smoking, Tobacco control, China

## Abstract

**Background:**

Although there are several studies to investigate the smoking behaviors among rural-to-urban Chinese migrants, no study has focused individually on this population in Shanghai. This study was performed to estimate the prevalence and identify the determinants of tobacco smoking among rural-to-urban migrants in Shanghai.

**Methods:**

In this cross-sectional study, multi-stage quota sampling was used to select 5,856 rural-to-urban migrants aged 18 years or older from seven districts in Shanghai between July and October 2012. A structured questionnaire was administered to assess smoking knowledge, attitude, behavior and demographic characteristics. Mental health was assessed by the self-reported SCL-90. Multiple logistic regression analysis was used to determine the risk factors of smoking behavior.

**Results:**

A total of 5,380 of the 5,856 migrants enrolled completed the questionnaire, among whom 45.0% of male and 2.0% of female participants reported current smoking. Multivariate analysis revealed current smoking in female migrants to be significantly associated with working at construction (OR, 8.08; 95% CI, 1.80-36.28), hotels/restaurants (OR, 5.06; 95% CI, 1.68-15.27), entertainment sector (OR, 6.79; 95% CI, 2.51-18.42), with monthly income > 3500 yuan (OR, 2.69; 95% CI, 1.21-5.98), number of migratory cities of 2 (OR, 2.39; 95% CI, 1.23-4.65), and SCL-90 total score > 160 (OR, 2.03; 95% CI, 1.03-3.98), while the male migrants working at construction (OR, 1.30; 95% CI, 1.04-1.62), entertainment sector (OR, 1.86; 95% CI, 1.36-2.56), being divorced/widowed (OR, 2.20; 95% CI, 1.02-4.74), with duration of migration of 4 or more than 4 years (OR, 1.42; 95% CI, 1.06-1.91), number of migratory cities of 3 or more than 3 (OR, 1.42; 95% CI, 1.13-1.80), and SCL-90 total score > 160 (OR,1.39; 95% CI, 1.07-1.79) showed an excess smoking prevalence.

**Conclusion:**

Migration lifestyle and mental status were associated with current smoking behaviors. The identifications of risk factors for current smoking may help to target health promotion interventions.

## Background

The World Health Organization (WHO) has estimated that smoking causes about 4 million deaths worldwide each year. This number is expected to exceed 8 million by 2030 [1]. More than 80% of these tobacco-attributable deaths will occur in the developing countries [1]. China is the largest consumer of tobacco in the world, producing a heavy burden of smoking-related diseases. According to the Global Adult Tobacco Survey conducted in 2010, 301 million adults in China are current smokers and 72.4% non-smoking adults are exposed to secondhand smoke in a typical week [2]. Approximately 1 million people die from smoking-related diseases annually and it is expected that this death number will reach 2 million a year by 2025[3]. The treatment of smoking-related diseases also accounts for almost 6% of the total medical expenses in China [4].

More than three-quarters of the population of China live in rural areas [5]. As a result of rapid industrialization, China has rapidly urbanized. More and more rural migrant workers move across counties or provinces to find new jobs. Rural-to-urban migrant workers are a special group in China. The term “rural-to-urban migrant” in China refers to farmers who move from rural to urban areas seeking employment and better living standards, without obtaining permanent urban residency (“hukou”) [6]. According to the National Bureau of Statistics, the number of rural-to-urban migrant workers increased from 121 million in the year 2000 to 262 million in 2012 [7], and will continue to grow. Shanghai is one of the largest metropolitan areas in China, with a population over 23 million [8]. As China’s financial and economic center, Shanghai is a popular destination for rural migrants from the inner provinces [9]. During the past two decades, the number of rural-to-urban migrant workers living in Shanghai has increased greatly, with the estimated population of 9 million migrant workers from across China, accounting for more than one-third of Shanghai’s total population in 2010 [8].

Migration from rural to urban areas necessitates alterations in social status and living conditions that result in behavioral adaptations to urban life. Migrant workers are more likely to take riskier jobs than Shanghai natives. These jobs involve heavy labor and low income, such as working in the manufacturing, construction industry or service sector. Migrants usually undertake more physical workload, longer working hours, but receive less labor protection [10]. The stress induced by migration itself, unstable living situations and poor working conditions, is likely to increase the risk of substance abuse, including smoking, in this population [11]. Several studies suggest that rural-to-urban migrants have an excess smoking prevalence [12,13]. However, the relationship between smoking behavior and rural-to-urban migration in China has not been well characterized. The objective of our study was to investigate smoking behavior, knowledge regarding smoking, and attitude toward smoking in rural-to-urban migrant workers living in Shanghai. We aimed to identify demographic characteristics, migrant status and psychological conditions associated with smoking in order to aid the design of an effective public health intervention strategy for tobacco control.

## Methods

### Study design and sampling

A cross-sectional survey of rural-to-urban migrant workers in Shanghai was conducted. Between July and October 2012, rural-to-urban migrants were recruited by using a multistage systematic sampling procedure. In stage 1, seven of the seventeen districts of Shanghai were randomly selected (Changning, Pudong, Putuo, Yangpu, Xuhui, Jiading, and Qingpu). These districts represented the inner-city (Changning, Yangpu and Xuhui), suburban (Jiading and Qingpu) and urban fringe zone (Pudong and Putuo). In stage 2, one sub-district in each of the seven study districts was randomly selected (Cao hejing, Chang zheng, Xin jing, Yin hang, San lin, Huang du, and Hua xin). In stage 3, a quota-sampling procedure was applied to recruit the participants. According to the employment of migrant workers reported by the National Bureau of Statistics 2011 [14], six occupational clusters employed 83.7% of the rural-to-urban migrants in East China, including manufacturing, construction, hotels and restaurants, domestic service, wholesale and retail, and entertainment (such as barbers, beauty salons, bath houses, and night clubs). These six occupational clusters were selected as the sampling frame and worksites that employed migrants in seven sub-districts were used as the sampling units. Employers (or workplace managers) at sampling units were contacted for permission to conduct the survey on their premises. A total of 112 worksites (employees varied between 100 in some small-sized worksites to more than 1000 in those large factories) were selected from seven sub-districts according to the occupational cluster and then 20% of these worksites were randomly sampled by type. In stage 4, eligible participants were selected at the sampling units. An eligible participant was defined as an individual who was registered at a rural residence, had been working in Shanghai for at least 6 months without obtaining permanent residence, and was aged 18 or above. The number of participants was approximately proportionate to the overall distribution of the migrant population by occupational cluster. The aim and content of the survey were explained to all participants, and all provided written informed consent. Approval for this study was obtained from the Ethics Committee of Zhongshan Hospital, Fudan University.

### Survey methods and data collection

A self-administrated questionnaire was designed to collect data. Questionnaires were pilot-tested for comprehension and appropriateness of language prior to the main study. Participation was voluntary and anonymous and the questionnaire was completed individually in a private interview room to protect the participant’s confidentiality. General practitioners supervised the accomplishment of questionnaire by the study subjects themselves. Assistance was provided to those with difficulties in completing the questionnaire, owing to an educational deficit. The questionnaire required approximately 35 minutes to be completed. General practitioners checked the returned questionnaires for completeness on-site. All data collectors received training in the research methods prior to commencement of the survey. In addition, supervisors from Zhongshan Hospital observed 10% of the data collection process from each general practitioner to ensure the data quality.

The survey collected the following information: (1) Socio-demographic characteristics including age, gender, marital status, educational level, and income; (2) Employment and migratory history, including type of work, hours worked per day, years as a migrant worker and number of cities resided in since migrating to an urban area; (3) Smoking-related behavior including current smoking behavior, exposure to secondhand smoke, and intention to quit smoking; and (4) Knowledge and attitude toward anti-smoking policies. The participants were classified according to the definition in the 2002 National Survey [15]: 1) Participants that responded positively to the question “Have you ever smoked more than 100 cigarettes?” were categorized as smokers; 2) Participants reporting smoking within the 30 days preceding the interview were categorized as current smokers. The current smokers group comprised both daily and occasional smokers; 3) Participants reporting smoking 20 or more cigarettes per day were categorized as heavy smokers; and 4) Non-smoking participants reporting exposure to secondhand smoke for at least 1 day per week and over 15 minutes per day were categorized as passive smokers. Psychological status was assessed using a Chinese version of the symptom checklist-90 (SCL-90), a self-reported multidimensional inventory designed to screen for a broad range of psychological problems and symptoms of psychopathology, which includes 90 questions, each scored on a 5-point Likert scale. In China, the SCL-90 has previously been used to assess mental health among different populations, including migrant workers [16]. According to the baseline score of the SCL-90 in China, participants with SCL-90 total scores exceeding 160 were classified as mentally unhealthy [17].

### Statistical analysis

All data were double-entered independently and validated using EpiData version 3.1 (EpiData Association, Odense, Denmark). All statistical analyses were performed using SPSS version 20.0 (IBM, Chicago, IL, USA). The socio-demographic characteristics, employment and migratory history, smoking-related behavior, knowledge and attitude toward anti-smoking policies, and psychological status were presented as the frequency distributions, percentage, mean and standard deviation. The Chi-square test was performed to examine the associations between the smoking prevalence and the socio-demographic characteristics, migratory history, working conditions, and psychological status. The smoking-related knowledge and attitudes among current and non-current smokers were also tested by Chi-square test. Multivariate analysis with logistic regression model was applied to identify independent variables associated with current cigarette smoking, from which adjusted odds ratios (OR) and 95% confidence intervals (CI) were calculated. All the above smoking-related factors, including the socio-demographic characteristics (age, marital status, educational level, and income), employment and migratory history (type of work, hours worked per day, years as a migrant worker and number of migratory cities), and psychological status were analyzed in terms of smoking status (defined the current smokers = 1; non-current smokers = 0). Because of the great gender difference in smoking number in this study, regression model predicting determinants associated with current smoking behaviors was stratified by gender. P-value of less than 0.05 (2-tailed) was considered statistically significant.

## Results

### Characteristics of the study population

Of a total of 5,856 eligible participants, 5,380 completed the questionnaire, with an overall response rate of 91.9%. Participants ranged from 18 to 65 years of age, with a mean age of 34.3 ± 10.5 years. Roughly half (50.4%) of the participants were male (Table 1). The average age of female participants (33.2 ± 10.0 years) was lower than that of males (35.3 ± 10.9 years).

**Table 1:** **Current smoking prevalence by demographics, migratory history, working conditions and psychological status**

**Variable**	**Total (n)**	**Current smokers**		***χ*** ^***2***^	**P**
		**n**	**%**		
**Gender**					
Female	2671	54	2.0	1376.84	< 0.01
Male	2709	1220	45.0		
**Age (years)**					
<25	1252	293	23.4	13.69	<0.01
25-34	1569	324	20.7		
35-44	1503	384	25.5		
≧45	1056	273	25.9		
**Employment sector**					
Manufacturing	2459	590	24.0	284.11	<0.01
Construction	740	324	43.8		
Hotels/restaurants	384	81	21.1		
Domestic service	569	43	7.6		
Wholesale/retail	628	86	13.7		
Entertainment	600	150	25.0		
**Marital status**					
Married	3937	902	22.9	11.85	<0.01
Single	1315	329	25.0		
Cohabiting	79	23	29.1		
Divorced/widowed	49	20	40.8		
**Education**					
Primary school or lower	984	173	17.6	53.75	<0.01
Junior high school	2731	622	22.8		
Senior high school	1297	394	30.4		
College	368	85	23.1		
**Monthly income (RMB: yuan)**					
≤2500	2967	512	17.3	162.35	<0.01
2501-3500	1661	492	29.6		
>3500	752	270	35.9		
**Duration of migration (years)**					
<1	1112	237	21.3	18.74	<0.01
1-3	1542	329	21.3		
4-5	724	172	23.8		
>5	2002	536	26.8		
**Number of migratory cities**					
1	2715	511	18.8	182.95	<0.01
2	1299	281	21.6		
3	696	204	29.3		
4	239	79	33.1		
≧5	431	199	46.2		
**Hours worked per day**					
<8	79	8	10.1	50.30	<0.01
8-10	3081	748	24.3		
11-12	1247	355	28.5		
>12	973	163	16.8		
**SCL-90 total score**					
≦160	4800	1109	23.1	8.17	<0.01
>160	580	165	28.4		

Approximately half of the respondents only had completed junior high school (50.8%). The majority (73.2%) of participants were married. About half (45.7%) of the participants worked in manufacturing, 13.8% in construction, 11.7% in wholesale/retail, 11.2% in entertainment, 10.6% in domestic service, and 7.1% in hotels/restaurants. The majority (62.8%) reported their duration as a migrant to be 5 years or less. Almost 50.5% of the participants reported having migrated to only one city, while 8% reported having been in five or more cities (Table 1). Most of the participants (89.2%) presented with healthy psychological status (SCL-90 total scores≦160) (Table 1).

### Smoking behavior, knowledge and associated factors

Among the 5380 migrant worker participants, 1427 (26.5%) were smokers and 1274 were classified as current smokers, resulting in a current smoking prevalence of 23.7% in our sample. Significantly fewer female participants were current smokers than the male participants (2.0% *vs.* 45.0%). Among the 1274 current smokers, 11.2% were heavy smokers, while 2004 participants (50.7%) in 3953 non-smokers were reported to be passive smokers because of exposure to secondhand smoke for at least 1 day per week and over 15 minutes per day. Table 1 shows that current smoking prevalence varied by various variables.

In addition, we also investigated the smoking behavior of current smokers: the mean cumulative smoking time reported by current smokers was 10.7 ± 9.0 years (range: 1 to 48 years); current smokers consumed an average of 13.0 ± 9.0 cigarettes per day; approximately 55.2% of current smokers reported smoking at their workplace and 58.8% reported previous attempts to quit smoking (Table 2). Common reasons for trying to quit smoking were reported to be awareness of the dangers of smoking to health (65.7%), family member pressure (18.3%) and economic factor (11.2%). Nearly two-thirds of current smokers reported that they would reduce cigarette consumption or quit smoking in the coming year.

**Table 2:** **Smoking behavior among current smokers**

**Variable**	**n**	**%**
**Cumulative smoking time (years)**		
≦5	494	38.8
6-10	365	28.6
11-20	269	21.1
>20	146	11.5
**Number of cigarettes smoked per day**		
≦10	622	48.8
11-20	509	40.0
21-30	105	8.2
≧31	38	3.0
Expenditure on smoking per month (RMB: yuan)*		
<50	267	21.1
50-100	253	20.0
>100	745	58.9
**Location for smoking**		
Smoking at home*		
YES	919	80.6
NO	221	19.4
Smoking at workplace*		
YES	553	55.2
NO	448	44.8
**Smoking at public place**		
YES	233	18.3
NO	1041	81.7
**Past-year quit attempts**		
YES	749	58.8
NO	525	41.2

*Sum of the respondents for different categories in Table 2 was not identical because of missing data on the corresponding variables.

Of all the participants, about 66.3% participants were against smoking, 28.8% expressed neutral attitudes, and only 4.9% expressed positive attitudes toward smoking. The vast majority of those participants (82.2%) stated that smoking was very harmful to health, while 17.8% of migrant workers thought smoking was harmless to health. More than 60% of the participants (67.7%) had a positive attitude toward a ban on smoking in public places, whereas 14.5% had a negative attitude to such a ban (Table 3). Smoking-related knowledge and attitudes differed significantly between current smokers and non-current smokers (Table 3): 86.4% of non-current smokers were aware of the harm of smoking, in comparison to 68.6% of current smokers (P < 0.01); 72.7% of non-current smokers were in favor of a ban on smoking in public places, whereas only 51.8% of smokers supported such a ban (P < 0.01).

**Table 3:** **Smoking-related knowledge and attitudes among current smokers and non-current smokers**

**Knowledge and attitude**		**Current smokers (n, %)**	**Non-current smokers (n, %)**	***χ*** ^***2***^	**P**
**Knowledge**					
Smoking is harmful to health	Yes	874 (68.6)	3549 (86.4)	211.41	<0.01
	No	400 (31.4)	557 (13.6)		
Passive smoking is harmful to health	Yes	900 (70.6)	3514 (85.6)	147.29	<0.01
	No	374 (29.4)	592 (14.4)		
**Attitude**					
Attitude toward smoking	Negative	280 (22.0)	3290 (80.1)	1573.07	<0.01
	Neutral	787 (61.8)	762 (18.6)		
	Positive	207 (16.2)	54 (1.3)		
Attitude toward smoking bans in public places	Negative	228 (17.9)	552 (13.4)	223.27	<0.01
	Neutral	386 (30.3)	570 (13.9)		
	Positive	660 (51.8)	2984 (72.7)		

### Multiple logistic regression analysis of determinants

The multiple logistic regression results stratified by gender are shown in Table 4. In the female-specific model, working at construction (OR = 8.08; 95% CI = 1.80 - 36.28), hotels/restaurants (OR = 5.06; 95% CI = 1.68-15.27), entertainment sector (OR = 6.79; 95% CI = 2.51-18.42), with monthly income > 3500 yuan (OR = 2.69; 95% CI = 1.21-5.98), number of migratory cities of 2 (OR = 2.39; 95% CI = 1.23-4.65), and SCL-90 total score > 160 (OR = 2.03; 95% CI = 1.03-3.98) significantly increased the likelihood of current smoking.

**Table 4:** **Multiple logistic regression analysis of determinants associated with current smoking by gender**

	**Model 1 (female)**							**Model 2 (male)**					
**Variable**		**Current smokers (n)**	**Non-current smokers (n)**	**cOR (95% CI)**	**P**	**aOR (95% CI)**	**P**	**Current smokers (n)**	**Non-current smokers (n)**	**cOR (95% CI)**	**P**	**aOR (95% CI)**	**P**
**Age (years)**	<25	26	641	1.00		1.00		267	318	1.00		1.00	
	25-34	22	835	0.65 (0.37-1.16)	0.143	0.97 (0.46-2.05)	0.927	302	410	0.88 (0.70-1.09)	0.244	0.71 (0.55-0.93)	**0.014**
	35-44	1	692	0.04 (0.01-0.26)	0.001	0.09 (0.01-0.74)	**0.026**	383	427	1.07 (0.86-1.32)	0.544	0.76 (0.57-1.02)	0.064
	≧45	5	449	0.28 (0.11-0.72)	0.009	0.58 (0.16-2.10)	0.408	268	334	0.96 (0.76-1.20)	0.698	0.73 (0.53-0.99)	**0.040**
**Employment sector**	Manufacturing	6	1099	1.00		1.00		584	770	1.00		1.00	
	Construction	3	90	6.11 (1.50-24.82)	0.011	8.08 (1.80-36.28)	**0.006**	321	326	1.30 (1.08-1.57)	0.006	1.30 (1.04-1.62)	**0.020**
	Hotels /restaurants	8	231	6.34 (2.18-18.46)	0.001	5.06 (1.68-15.27)	**0.004**	73	72	1.34 (0.95-1.88)	0.097	1.35 (0.95-1.92)	0.096
	Domestic service	3	426	1.29 (0.32-5.18)	0.720	1.54 (0.35-6.78)	0.566	40	100	0.53 (0.36-0.77)	0.001	0.56 (0.38-0.84)	**0.005**
	Wholesale/retail	1	403	0.46 (0.06-3.79)	0.466	0.69 (0.08-6.12)	0.740	85	139	0.81 (0.60-1.08)	0.146	0.84 (0.62-1.13)	0.248
	Entertainment	33	368	16.43 (6.83-39.51)	0.000	6.79 (2.51-18.42)	**0.000**	117	82	1.88 (1.39-2.55)	0.000	1.86 (1.36-2.56)	**0.000**
**Marital status**	Married	22	1955	1.00		1.00		880	1080	1.00		1.00	
	Single	28	603	4.13 (2.34-7.27)	0.000	1.61 (0.73-3.52)	0.236	301	383	0.97 (0.08-1.15)	0.686	0.88 (0.70-1.11)	0.286
	Cohabiting	3	41	6.50 (1.87-22.59)	0.003	1.10 (0.26-4.62)	0.899	20	15	1.64 (0.83-3.22)	0.153	1.40 (0.69-2.85)	0.356
	Divorced/widowed	1	18	4.94 (0.63-38.62)	0.128	2.49 (0.28-22.32)	0.415	19	11	2.12 (1.00-4.48)	0.049	2.20 (1.02-4.74)	**0.044**
**Monthly income (RMB: yuan)**	≤2500	19	1789	1.00		1.00		493	666	1.00		1.00	
	2501-3500	15	609	2.32 (1.17-4.59)	0.016	1.54 (0.72-3.27)	0.266	477	560	1.15 (0.97-1.36)	0.103	1.10 (0.92-1.31)	0.300
	>3500	20	219	8.60 (4.52-16.36)	0.000	2.69 (1.21-5.98)	**0.016**	250	263	1.28 (1.04-1.58)	0.019	1.13 (0.90-1.43)	0.292
**Education**	Primary school or lower	6	604	1.00		1.00		167	207	1.00		1.00	
	Junior high school	27	1354	2.01 (0.83-4.89)	0.125	0.61 (0.22-1.70)	0.345	595	755	1.42 (1.01-1.99)	0.042	0.93 (0.73-1.18)	0.550
	Senior high school	19	522	3.66 (1.45-9.24)	0.006	0.53 (0.17-1.62)	0.265	375	381	1.39 (1.04-1.85)	0.027	1.16 (0.88-1.51)	0.296
	College	2	137	1.47 (0.29-7.36)	0.640	0.30 (0.05-1.71)	0.176	83	146	1.73 (1.28-2.35)	0.000	0.73 (0.50-1.06)	0.101
**Duration of migration (years)**	<1	18	562	1.00		1.00		219	313	1.00		1.00	
	1-3	20	799	0.78 (0.41-1.49)	0.454	0.94 (0.47-1.88)	0.865	309	414	1.07 (0.85-1.34)	0.577	1.21 (0.95-1.53)	0.121
	4-5	8	356	0.70 (0.30-1.63)	0.410	1.45 (0.58-3.64)	0.433	164	196	1.20 (0.91-1.57)	0.194	1.42 (1.06-1.91)	**0.018**
	>5	8	900	0.28 (0.12-0.64)	0.003	1.11 (0.40-3.07)	0.845	528	566	1.33 (1.08-1.64)	0.007	1.76 (1.36-2.26)	**0.000**
**Number of migratory cities**	1	17	1480	1.00		1.00		494	724	1.00		1.00	
	2	23	690	2.90 (1.54-5.47)	0.001	2.39 (1.23-4.65)	**0.010**	258	328	1.15 (0.95-1.41)	0.162	1.17 (0.95-1.43)	0.138
	3	10	290	3.00 (1.36-6.62)	0.006	2.27 (0.97-5.30)	0.058	194	202	1.41 (1.21-1.77)	0.003	1.42 (1.13-1.80)	**0.003**
	4	1	83	1.05 (0.14-7.98)	0.963	0.71 (0.09-5.79)	0.750	78	77	1.49 (1.06-2.08)	0.021	1.52 (1.08-2.15)	**0.017**
	≧5	3	74	3.53 (1.01-12.31)	0.048	2.27 (0.59-8.80)	0.234	196	158	1.82 (1.43-2.31)	0.000	1.76 (1.32-2.28)	**0.000**
**Hours worked per day**	<8	1	61	1.00		1.00		7	10	1.00		1.00	
	8-10	40	1475	1.65 (0.22-12.23)	0.622	2.22 (0.28-17.48)	0.447	708	858	1.18 (0.45-3.11)	0.740	0.79 (0.28-2.18)	0.642
	11-12	8	490	1.00 (0.12-8.10)	0.997	1.59 (0.18-13.40)	0.674	347	402	1.23 (0.46-3.27)	0.674	0.74 (0.27-2.08)	0.570
	>12	5	591	0.52 (0.06-4.49)	0.549	1.64 (0.17-15.55)	0.664	158	219	1.03 (0.38-2.77)	0.952	0.74 (0.26-2.10)	0.575
**SCL-90 total score**	≦160	39	2338	1.00		1.00		1070	1353	1.00		1.00	
	>160	15	279	3.22 (1.75-5.92)	0.000	2.03 (1.03-3.98)	**0.041**	150	136	1.40 (1.09-1.78)	0.008	1.39 (1.07-1.79)	**0.012**

cOR, crude odds ratio; aOR, adjusted odds ratio. P values in bold are less than 0.05, indicating the statistical significance.

The male-specific model showed that migrants working at construction (OR = 1.30; 95% CI = 1.04-1.62), entertainment sector (OR = 1.86; 95% CI = 1.36-2.56), being divorced/widowed (OR = 2.20; 95% CI = 1.02-4.74), with duration of migration of 4 or more than 4 years (OR = 1.42; 95% CI = 1.06-1.91), number of migratory cities of 3 or more than 3 (OR = 1.42; 95% CI = 1.13-1.80), and SCL-90 total score > 160 (OR = 1.39; 95% CI = 1.07-1.79) were more likely to be current smokers.

A higher age appeared to be protective against smoking for female (OR = 0.09; 95% CI = 0.01-0.74) and male migrants (OR = 0.73; 95% CI = 0.53-0.99). Furthermore, the male migrants working for domestic service showed a low smoking prevalence (OR = 0.56; 95% CI = 0.38-0.84).

## Discussion

This present study was believed to be the first study to individually investigate the smoking habits of rural-to-urban migrant workers in Shanghai. A total of 5,380 rural-to-urban migrant workers in 7 districts of the central city, urban fringe zone and suburbs of Shanghai were enrolled, in whom 45.0% of male and 2.0% of female migrant workers reported current cigarette smoking. The gender difference of smoking prevalence in our sample was similar with the previous studies [18,19]. However, our current smoking prevalence of male seemed to be slightly lower than the estimate for a Beijing sample (51.7%) [12] and three cities sample (Chengdu, Shanghai and Beijing) (51%) [20]. The fraction of female smokers in our sample was also significantly lower than that reported by Chen *et al*. for rural-to-urban migrants in Beijing almost ten years ago (2% *vs* 10.9%) [12]. These variations in reported rates of smoking in this study with the study by Chen *et al*. [12] and Yang *et al*. [20] may be attributed to the different study location, sampling frames, and demographic characteristics of the population enrolled [18]. In addition, the prevalence of current smoking for males in our study was also observed to be lower than the national prevalence reported in the 2010 Global Adult Tobacco Survey (GATS) (52.9%) [2] and the prevalence for general male population in Shanghai (54.8%) [21]. This might result from the lower income of migrants in cities. Migrants belong to the lower socioeconomic rank of cities and always be paid at a minimum wage compared with urban counterparts, causing the huge gap of wages between migrants and workers with an urban residency [22]. According to the Shanghai Bureau of Statistics in 2012 [23], the average monthly income of residents was 4692 yuan, which was much higher than that of the migrants in our study. The economic factor may have reduced the likelihood for many migrants to engage in smoking behaviors in urban areas [24]. Moreover, the main goal of migrants leaving home was to earn money which also makes them more likely to lessen or abandon their smoking behaviors to save more money and send money home [19], thus leading to the lower prevalence of smoking. Previous studies also showed that tobacco smoking prevalence was lower in migrant men than that in the urban and rural residents [19,24].

Three groups of risk factors were found to be significantly associated with smoking in our study. First, occupation was a determinant of smoking risks among rural-to-urban migrants. Female migrants working at construction sites, hotels/restaurants and entertainment venues had 8.8 times, 5.06 times and 6.79 times the likelihood to be current smokers compared with those working in factories. Similarly, the male migrants working at construction sites and entertainment venues had 1.3 times and 1.86 times the likelihood to be current smokers. The increased risk of smoking experienced by migrants working in the construction sector may be first attributed to this high stress job. Exposure to occupational hazards (e.g., chemicals and dust) is a typical job stressor for construction workers, which has been demonstrated to positively affect current smoking [25,26]. Next, construction is a high hazard but low-income occupation, and payment is often delayed or withheld [27]. This results in a lower socioeconomic status among construction workers and makes them more prone to smoking. Thirdly, the gender discrimination from supervisors and coworkers may be a reason for the higher odds of smoking among female construction workers than the male construction workers [25,28]. Thus, tobacco control intervention for this population should consider work-related occupational factors along with individual approaches [25].

The increased odds of smoking among migrant workers employed in hotels, restaurants and entertainment venues may be related to that particular workplace making them more exposed to smoking, resulting in a significantly higher likelihood of smoking [13]. Furthermore, branding and packaging of female specific cigarettes is an effective approach to recommend cigarettes to young women, leading to the substantially higher prevalence of current smoking among women who was exposed to female specific brand cigarettes than those who did not attempt female brands [29]. To prevent these young women from smoking, related regulations should be developed to limit the packaging and advertisement for female targeted cigarettes brands [13].

Second, our study also suggested that migratory history was positively associated with current smoking behavior. For male migrant workers, longer time living in an urban environment correlated with an increased risk of smoking. This may result from the stress caused by long-term separation from family and pressure to establish social relationships, as well as increased exposure to foreign cultures and urban cigarette marketing campaigns [12]. The number of cities they migrated through was also associated with elevated risk of smoking, which may be ascribed to the stress for adapting to new environments and circumstances and the high concern for the unstable living situations and employment opportunities [20]. This seemed to be more severe for male migrants because the more cities migrated, the male migrants were more susceptible to be current smokers.

Last, the regression model showed that migrant workers with unhealthy psychological status (SCL-90 total scores > 160) were at a higher level of risk for cigarette smoking. This supports previous evidence that psychosocial stress was an important risk factor for smoking among urban residents [30]. Navigating life in urban areas, instability of living and employment conditions, discrimination, and lack of social support also can induce a high level of psychosocial stress and high rates of mental problems for migrant workers [31], which in turn increase the susceptibility to cigarette smoking. Special prevention initiatives addressing the need to reduce exposure to psychosocial stress should target rural-to-urban migrants in order to help prevent smoking.

In addition to the above three crucial factors for female and male migrants, the female with high monthly income and the male being divorced/widowed may also be significantly related with the smoking prevalence. Female migrants who earned higher incomes may work in entertainment sectors such as night clubs and thus are more likely to be intoxicated by smoking [11]. Marital termination may lead to a loss of spousal support to buffer against stress and then contribute to increased smoking [32]. A higher age and working at domestic service appeared to be protective factors for smoking. This may be due to the higher wish to quit smoking for a participant with higher age owing to health concerns and the customer requirement for domestic service migrants.

Our findings provide some suggestions for the direction in which tobacco prevention strategies could be targeted to high risk populations of rural-to-urban migrant workers. In our study, 55.2% of current smokers reported their workplace as the location they smoked most often, while only 18.3% migrants reported smoking at public place. In 2010, the Shanghai People’s Congress Standing Committee issued the Smoking Control Legislation in Public Places and totally prohibited smoking in 13 types of public places [33]. The implementation of these regulations further makes smoking at public places decrease somewhat [34]. This might explain the lower smoking rate at public places. However, this legislation is not a comprehensive ban and there is still no mention in the ban of workplaces. Previous studies have demonstrated that restriction of smoking in workplaces is an effective method by which tobacco consumption and exposure to second-hand smoke can be reduced [35,36]. Our data suggests that smoke-free policies in workplaces, in addition to public places, should be broadened and strengthened in China. Integrated healthcare programs should be provided to the migrant population to help them identify relevant risk factors for cigarette smoking and better enable them to stop smoking. In addition, healthcare programs should address stress management as an important component of smoking cessation. This includes the identification of stress risk-factors and appropriate strategies to cope with stressors, which may further reduce smoking behavior.

This study has several limitations. Firstly, we cannot draw conclusions regarding causality and the point estimates as well as associated variance estimates are likely biased because of the cross-sectional design, and non-random, quota sampling selection of participants. Secondly, we studied residents of only one city and cannot generalize the identified risk factors to the overall population of rural-to-urban migrant workers. Thirdly, having utilized a self-reporting questionnaire, our data may suffer from an information bias caused by interviewer expectations. However, since smoking is widely regarded as a normal behavior in China, the self-report bias in smoking research has been previously reported to be minimal [37].

## Conclusion

Our study reports the smoking status and risk factors for smoking among a highly vulnerable population of rural-to-urban migrant workers in Shanghai. Our findings indicate a need for tobacco control among this rapidly growing Chinese subpopulation. We recommend implementation of comprehensive targeted interventions addressing risk factors for smoking. Further research into the smoking behaviors of rural-to-urban migrants in additional cities is required in order to determine whether similar factors may contribute to smoking behavior nationwide.
